# Are all students getting maximum value from bioscience practicals? Exploring the benefits of inclusive competence-based design

**DOI:** 10.1042/ETLS20253032

**Published:** 2026-03-05

**Authors:** Dominic C. Henri, Katharine E. Hubbard

**Affiliations:** 1 School of Environment and Life Sciences, University of Hull, HU6 7RX, U.K.; 2 Learning Enhancement and Academic Practice, Buckinghamshire New University, HP11 2JZ, U.K.

**Keywords:** competence, education, learning, practical work, teaching

## Abstract

Practical work is expensive, complicated to organise and often exclusionary. However, practicals are commonly considered a core component of research-based bioscience education. This is difficult to justify when the majority of bioscience students will not go into bioscience research. We therefore need to have a robust rationale for including practical work in bioscience curricula, and to maximise the value of all practicals for all students whether in the laboratory, field or computational space. In this opinion article, we propose a competence-based education model for bioscience practical work, seeing competence as the ability to integrate skills, knowledge, attitudes and connections between practical learning and the wider world. We argue that using a competence model allows us to focus on the true value of practical work, and therefore to design more inclusive and authentic learning experiences. We encourage bioscience educators to rigorously question the purpose of practicals in their programmes, and whether they can maximise their educational value through more deliberate competence-driven curriculum design.

Practical work is expensive, complicated to organise and often exclusionary. In the current Higher Education climate, institutions are increasingly focussed on balancing budgets within an unsustainable financial model. There is also increasing pressure on universities to address the needs of national industrial strategies, provide ‘value for money’ for students and the tax-payer, support equitable outcomes for all groups of students and to ensure students get good jobs after their studies. Science programmes feel these multiple pressures acutely, with multiple course closures in recent years reflecting the high cost of our disciplines [1]. Within science programmes, there is more and more scrutiny over the inclusion of practical work in curricula, which is increasing fears about a lack of practical experience in graduates [2]. While we intuitively recognise the importance of practical work within science qualifications, against this backdrop bioscientists need to be able to make a coherent argument about its purpose, whether these occur in the laboratory, field or computational environment. We therefore think it is timely to explore how we can maximise the value of practical teaching within the biosciences for all students.

## What is the unique value of practical work in the biosciences?

While we recognise practical work has intrinsic value, it is important to consider what the unique educational value of that practical work is. If the value could be achieved outside of the practical environment, running a practical becomes a very expensive and time-consuming way to deliver that educational benefit. The unique value of practical learning in undergraduate biology degrees appears understudied compared with other sciences and in secondary education. Within the related disciplinary context of Chemistry, Michael Seery systematically asks ‘what is unique about learning in the laboratory environment that cannot be achieved elsewhere?’ [3]. He concludes that the lab is uniquely placed as ‘the place to learn how to do chemistry’, interpreted primarily as the development of technical skill (e.g. pipetting technique) and broader scientific skills (e.g. observation, interpretation, experimental design). We see parallels with Seery’s argument in terms of practicals as the authentic experiential context for learning how to ‘do biology’. This is supported by multiple studies that find that educators primarily perceive the value of practical teaching to be where students learn how to ‘do science’ or possibly experience how to ‘be a scientist’ [4,5].

This opens up two important questions. First, what does it mean to ‘do biology’ in the current higher education context? And secondly, what is the value of ‘doing biology’ to contemporary bioscience students, who go into a variety of diverse and rewarding careers within and beyond the biosciences?

Historically, ‘doing biology’ has been associated with hands-on experimental or observational work in either the lab or field environment. Some will argue that the physical preparation and manipulation of materials and samples as an essential competency in its own right, developing key psychomotor skills. Increasing amounts of bioscience research leverage digital, computational, modelling and qualitative approaches, so hands-on experimental work is no longer a requirement for bioscience research. Physical manipulation of samples is therefore an essential competency for *some* types of bioscience research and analysis. This does not make it an essential competency for *all* bioscience undergraduates. It is also exclusionary to assume that only those who can perform physical tasks in the lab or field can be biologists. ‘Doing biology’ is no longer restricted to the physicality of the lab or the field context, and we should embrace this as educators.

We also argue that ‘doing biology’ is much more than experimental manipulation and/or observation [4]. We view practicals as experiential learning that can act as a gateway to thinking and feeling like a biologist, i.e. the development of a biologist mindset. The unique opportunity to ‘be a biologist’ during active, experiential and authentic practicals can develop student logical and critical thinking, problem solving, ethical awareness, persistence and resilience as well as scientific identity.

It is also important to recognise that the majority of bioscience undergraduates find employment not within research or science industries, but in a diverse range of rewarding careers including policy, education, science communication, finance and in the broader economy [6,7]. Practical learning must have value to these students as well. If we position the unique value as the specific protocols and methods learned, then it is difficult to justify the inclusion of practical work for students who will never use these techniques. However, if we re-position the value as an experiential gateway developing a biologist mindset, we can support students to apply this insight into multiple career destinations and contexts. We often fail to purposefully design our practicals through these lenses, focussing instead on particular techniques or phenomena for students to observe [5]. So, how do we design practicals to maximise benefit for as many students as possible, and therefore fully justify their resource intensive nature?

## Maximising the value of practicals through competence-based education

Competency is a concept that helps to reposition educational value. Competence-based education (CBE) emphasises what students can do (or be) as the outcome of a learning experience, rather than just what they know [8–10]. The popularity of competence-based education has risen concurrently with multiple educational philosophies designed to decentre knowledge as the *de facto* outcome of Higher Education (e.g. Graduate Attributes, the 4th Industrial Revolution). Shared among these philosophies is a desire to centre curricula around authentic, experiential learning opportunities that offer learning outcomes that are valuable for a broad range of students’ aspirational futures.

**Figure 1:**
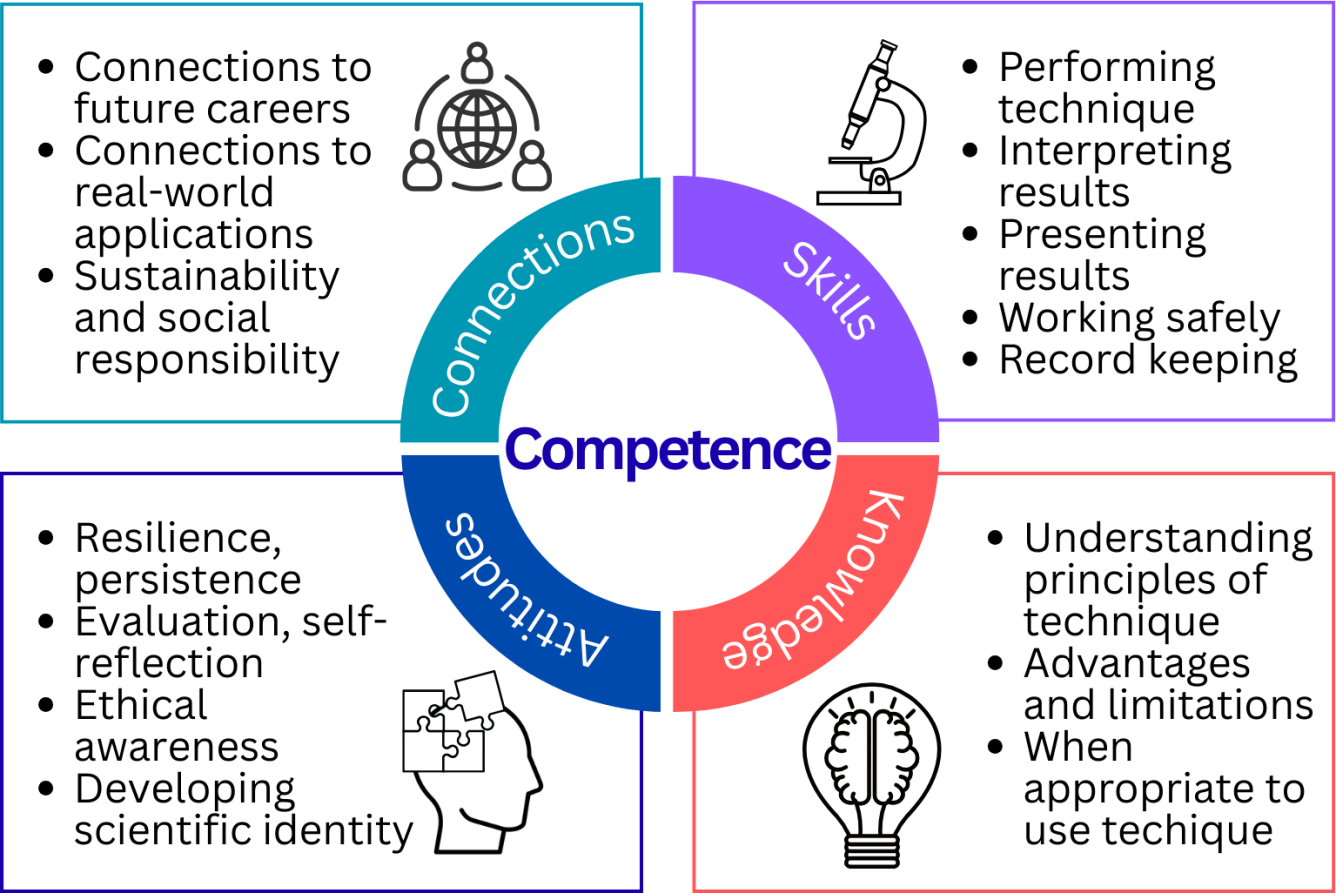
A proposed competence model for bioscience practical work.

There are multiple competence frameworks and no single definition of competency, but all define multiple goals of a learning experience, including skills, knowledge and attitudes [11]. We argue that it is also essential for students to make connections between their learning and the wider context, be that connections to future careers or global issues. Crucially, competence is to bring together these different dimensions within a disciplinary context (Figure 1). You cannot be fully competent if you can demonstrate the skills but cannot make the connections between these skills and the wider world, or have the self-awareness to evaluate if you have used the skills correctly or selected ethically appropriate methods.

So how does a competence-based education approach help us maximise the value of bioscience practical learning? Fundamentally, we need to zoom out from the details of particular methods and techniques, but to think about what practical work is ultimately for and how we use it to achieve the purposes of learning. We define the core competence of practical work as ‘using scientific approaches to create a rigorous explanation of the world around us’, which can be encapsulated by four domains of learning outcomes:

1. Knowledge: understanding what the scientific approaches are, how they work, advantages and limitations, and to recognise the positionality of that knowledge.
2. Skills: being able to select, perform, interpret and document the results of the scientific approaches.
3. Attitudes: developing an ethical, rigorous, iterative and empirical approach to building understanding, and the resilience required to deal with the set-backs and failures inherent to empirical methods.
4. Connections: understanding how these approaches are used within the real-world context, working within legislative and regulatory contexts, and linking approaches to student aspirational futures.

Those familiar with other educational frameworks may see parallels with our four domains. For example, Bloom’s Taxonomy includes cognitive, affective and psychomotor domains [12], which partially align with knowledge, attitudes and skills in our framework. Our framework also partially maps to other conceptualisations; for example, Hodson [13] defines four science-related learning goals of learning science (Knowledge), learning about science (Attitudes), doing science (Skills) and learning to address socio-scientific issues (Connections) [13].

## How could this competence model work in practice?

We argue that to get the best value out of practicals, we should optimise for all four domains of the framework as much as possible, for all students. Table 1 illustrates this for a standard 1st year undergraduate PCR laboratory practical, assessed through a pre-laboratory quiz and a structured question write-up. As PCR labs are expensive and complicated to run, instructors typically minimise opportunities for failure by using a tightly defined protocol and in having a back-up set of results for students to use if things do ‘go wrong’. However, this restricts opportunities for students to develop a more holistic understanding of PCR as a method and the scientific process more generally.

**Table 1:** Competence-based redesign of a PCR practical to maximise value

Domain of competency	Original design	Rating of original design	Revised model to maximise all aspects of competency
Knowledge	Students complete short pre-lab on theory of PCR, and structured question write-up	High	No change
Skills	Students follow a PCR protocol with pre-prepared primers, samples and controls in a whole day lab	Medium	Practical designed in two phases (i) computational design of primers and PCR conditions and (ii) PCR set-up and gel electrophoresis. Students are given the choice of which to do and/or write-up, or work in teams to complete the whole process. Documentation of practical work (e.g. lab book, risk assessment) forms part of assessment
Attitudes	Students have minimal opportunity to reflect on their learning, or to develop problem-solving skills as instructor-led design has minimised opportunities for failure	Low	Students are given a second practical session to iterate the methodology based on which aspects worked/didn’t. For example, If the PCR reaction fails, students are given the opportunity to troubleshoot and repeat. Response to problem solving components forms part of assessment
Connections	Students are focussing on specialist knowledge and skills with minimal connections to real world applications or careers	Low	PCR practical is situated in the context of a crime scene (e.g. human or wildlife forensics) with explicit links to external collaborators. The assessment encourages critical discussion of the reliability and application of PCR-based evidence and ethics

Applying our competency framework allows for instructors to significantly increase the value of the lab, without substantively changing the core experiment (Table 1). Our design uses the same core PCR reaction, but actively builds in opportunities for students to contextualise their learning, and to learn meaningful lessons from failure (a core aspect of developing a scientific attitude).

We recognise that this approach can come with resource implications. The example above requires repurposing learning time to accommodate a repeat lab, additional complementary workshops and a process-based assessment strategy. Where resources are limited, the computational component might be more appropriate for iteration, with students able to complete this in their own time with no need for specialist equipment or technical support. However, we can also ask what could be removed from the curriculum to accommodate a more valuable approach to practical design. If lab time is a limiting factor, educators could consider reducing the number of different practicals to maximise the value of one practical through including opportunities to repeat, problem-solve and iterate experimental work. We argue that the repeat practical that includes a problem-solving element has significantly greater educational benefit than introducing students to yet another technique they may never use again.

## Ensuring the value of practicals is inclusive to all students

All bioscience students should experience an inclusive education. However, disabled and pregnant individuals often find themselves excluded from practical work, either on the basis of physical capability or (often flawed) arguments based on health and safety. Adopting a competence-based approach can help to shape a more inclusive approach.

We first distinguish between competence-based education and competence standards, as these have different meanings under UK equality law [14]. We recognise that bioscience educators work under multiple legal frameworks, but see the distinction as having wider value in terms of shaping approaches to inclusion. A competence standard is legally defined as *an academic, medical or other standard applied for the purposes of determining whether or not a person has a particular level of competence or ability* [UK Equality Act, 2010]. All students must be able to meet the competence standard; however, reasonable adjustments can be applied to the mode of assessment used to demonstrate that standard. Competence standards must be objectively justified and are typically defined by Professional, Statutory and Regulatory Bodies (PSRBs), particularly in healthcare disciplines. They are most commonly present because they define some knowledge, skill or attitude that is considered essential to a profession that the educational programme is training the student to be able to perform competently (e.g. a fit for practice qualification).

These types of standards are rare within the biosciences, particularly because no two bioscience programmes are the same and they are rarely training every student for a single profession with direct legal oversight. Given this diversity of curricula, we cannot objectively justify being able to perform any specific research method as essential to getting a bioscience qualification. However, we can apply a competency-based education approach as articulated above to identify the most important aspects of the practical learning experience.

## Applying the competence model in Practice

Let us return to the PCR lab to illustrate how the competency-based approach aids inclusive design (Table 1). PCR may be a foundational molecular biology technique, and essential in some lab-based life-science jobs, but performing the technique cannot be considered a competence standard for all biologists. So how can a competency model help us avoid excluding students who cannot perform the technique as well as maximise the value of the practical for every student?

Focussing on the four competence-based outcome domains helps to establish the overall value of the activity. Practical competence is not just to be able to prepare samples and set up an electrophoresis gel, but to use appropriate scientific approaches to understand the world, in this case through amplification of DNA using primers designed against a specific target. Students are equally able to demonstrate this competency through computational design of primers, testing their efficacy using *in silico* approaches and predicting the PCR conditions required. In our design above, students could do either the computational primer design practical or the wet lab practical and still meet the competency. Students could do both, or just one component. To introduce elements of collaboration, students could be placed into groups where half did the computational design and then half tested the primers in the lab, and communicated between the two teams to optimise the design.

This design creates an inherently authentic and inclusive option for those unable to engage with the wet lab work, without the need for individual reasonable adjustments. We are not arguing for the exclusion of any learner from the practical environment, and instructors should still make reasonable accommodations to ensure that all can safely and meaningfully participate in laboratory and field work [15,16]. However, where this is genuinely not possible, our model ensures the development of equivalent scientific competencies through a high quality authentic alternative that is proactive to the needs of a diverse student body.

## Implications for curriculum design

Thus far, we have considered the design of an individual practical to maximise value for all. We argue that bioscience educators need to think more carefully about when and where practical work is positioned within the curriculum. We are not arguing for the removal of hands-on practical work and value its role in developing psychomotor skills as well as developing scientific identity. However, we are arguing for more deliberate curriculum design choices, including considering when and where hands-on practical work is the most effective activity to support student learning within programmes. Where we place assessed practical work in core modules for all students, we have an ethical responsibility to ensure that all can engage with it, and that the practical work is positioned in a way that connects it to as many students’ aspirational futures as possible.

We also encourage readers to consider whether their curriculum is sufficiently focused on the right number and type of learning outcomes or competencies. Traditional STEM curricula are known to be ‘overly full’ [17] and overly fact-based [18]. The competency framework can help here as a tool to identify whether learning outcomes are equally distributed across all domains, or over-prioritise knowledge. Particularly, we should be mindful of the burden of curriculum creep, where over time degrees require mastery of increasing and increasingly complex concepts, mechanisms and research findings. While curricula should be up to date and research informed, cramming in more and more specialist examples or techniques actively curtails opportunities to develop other aspects of student learning. We argue that the resources required to embed Attitudes and Connections into practical learning (which have value to the broadest range of student futures) could come from those currently invested into teaching, learning and assessing disciplinary knowledge (which has value to the narrowest range of student futures). Below we suggest some design considerations that can be used to enhance the value of practical learning experiences in combination with the competence framework.

### 1. Look beyond individual, specific, physical methods

Practical education can become more inclusive by thinking about alternative methods of observing and investigating scientific phenomena. Complementing physical tasks with simulated, digital or computational tasks, which can also replicate authentic scientific inquiry, broadens the scope of practicals inclusively for all. Learning to use a specific method is likely to be less broadly valuable than the opportunity to experience ‘doing science’.

### 2. Actively connect practical learning to student aspirational futures

Asking students to develop practical skills in isolation from their application in the real world undermines student perception of the value of practical competence and limits their ability to transfer those competencies to new contexts. We recommend working with external experts (e.g. industry, local government) to design practical teaching and assessment that mirrors how these competences are used in authentic contexts relevant to student futures. To maximise the ‘connections’ domain, it can be especially valuable to draw on how these experts use practical work to address socio-scientific challenges. This approach also gives the opportunity to mirror industry-relevant practices including robust documentation practices, budgeting or risk assessment.

### 3. Make all four competence-based domains explicit learning outcomes

Educators need to ensure that course materials highlight the holistic benefits of practical learning beyond the development of practising specific scientific methods or even scientific inquiry. There is no reason to assume that students will implicitly develop beneficial attitudes and connections, so these must be made explicit and actively supported. Using reflective activities and assessments is common practice for achieving this in profession-orientated degrees, and we advocate for their use in the biosciences to help students realise the full extent of their development.

### 4. Design richer specialist practical experiences

Many programmes have practicals tied to core theoretical modules, giving all students only limited hands-on experience. We would advocate for high-quality optional intensive practical modules that are clearly connected to aspirational futures. These become rich learning experiences for those students who really value these technical competencies and want to develop them for future career development. Students who do not want to undertake this intensive practical training could be offered other high quality options such as professional consultancy projects or science communication that develop alternative competencies. An intensive practical model allows instructors to give the time and focus that hands-on skill development actually needs and deserves. A structured intensive practical-based module can also give students more autonomy over the experimental work they perform, more opportunities to engage in the messy unpredictable nature of scientific inquiry, design and optimise their own experiments, learn from failures, reflect on their learning and make authentic connections to industrial applications of practical work.

## Conclusions

In a contemporary resource-stretched world where most students won’t become research scientists, practicals have to be about more than learning how to pipette or setting up a transect. We absolutely should include practical work in bioscience curricula as a key aspect of disciplinary experiential learning. Adopting a competency-based approach has significant potential for maximising the broader value of that practical work. While there are multiple definitions of competence, our model emphasises integration of knowledge, skills, attitudes and connections to building a scientific understanding of the world, and supports inclusive practice. To be effective, creating these links should be an active design intention of the educator, not a tangential or accidental benefit [5]. If we are setting up a practical, we have a responsibility to ensure that time, effort and expense are fully justified. In our experience, a competency-based approach can unlock the full potential of a practical, maximising the value for all. We challenge bioscience education colleagues to rigorously question the purpose of practicals in their programmes, and to make more deliberate curriculum design decisions to maximise the value of practical-based learning experiences.

## Open Access

Open access for this article is provided via our Subscribe to Open programme, made possible through the support of our subscribing institutions. Learn more here: https://portlandpress.com/pages/open_access_options_and_prices#conditional

## Abbreviations

CBE: competence-based education
PSRBs: Professional, Statutory and Regulatory Bodies
